# The impact of egg-based supplementation on child malnutrition in sub-Saharan Africa: a meta-analysis of randomised controlled trials

**DOI:** 10.1017/jns.2026.10097

**Published:** 2026-05-06

**Authors:** Legese Petros, Alemneh Kabeta Daba, Abadi Gebre Mezgebe, Afework Mulugeta

**Affiliations:** 1 School of Nutrition and Food Science and Technology, Hawassa University College of Agriculture, Ethiopia; 2 Central Ethiopia Public Health Institute, Ethiopia; 3 School of Nursing, Hawassa University College of Medicine and Health Sciences, Ethiopia; 4 Nutrition and Dietetics, School of Public Health, Mekelle University College of Health Sciences, Ethiopia

**Keywords:** Children underfive, Egg-based supplementation, Growth, Meta-analysis, Randomised controlled trial, Sub-Saharan Africa

## Abstract

Malnutrition remains a major public health challenge in low- and middle-income countries and disproportionately affecting children under five. Eggs, given their high nutrient density and relative physical or economic accessibility, have been tested for their effect on improving nutritional outcomes in children under five. However, findings from scientific exercises to test the impact of egg-based trials on child growth have not been systhematically pooled and synthesised. Therefore, this meta-analysis aimed to synthesise evidence on the impact of egg-based interventions on the nutritional status of children underfive as determined by weight-for-height *Z*-score (WHZ), weight-for-age *z*-score (WAZ), and height-for-age *z*-score (HAZ). Research articles of randomised controlled trials published between 2013 and 2023 were identified through a comprehensive search of PubMed/MEDLINE, Web of Science, CINAHL, Embase, Science Direct, Google Scholar, and African Index Medicus data bases. Articles evaluated the effect of egg-based interventions against alternative diets, behaviour-change education, or no alternative intervention were included. Primary outcomes are WHZ, WAZ, and HAZ. Random-effects models were used to pool effect sizes (mean difference), and subgroup analyses and meta-regression explored sources of heterogeneity. Publication bias was assessed using funnel plots and Egger’s test. Seven studies involving 3673 children met the inclusion criteria. Egg-based intervention significantly improved WAZ (MD: 0.33; 95% CI: 0.11–0.55) and WHZ (MD: 0.30; 95% CI: 0.12–0.48). However, no significant effect was observed on HAZ (MD: 0.05; 95% CI: –0.05–0.14). It is figuredout that egg-based interventions can improve weight-related nutritional outcomes (WHZ and HAZ) among children underfive in sub-Saharan Africa, but not linear growth (HAZ).

## Introduction

Malnutrition is a common public health problem in the world, especially among children under the age of five years in low- and middle-income countries. As per the estimates of the UNICEF, the WHO, and the World Bank, there were 150.2 million children under 5 estimated to be stunted and 42.8 million wasted in 2025.^(1,2)^ These statistics underscore the urgent need for feasible food-based solutions to combat undernutrition and its multifaceted long-term consequences.

Egg supplementation or egg-based interventions refer to any nutritional or dietary strategies that include the promotion, provision, or supplementation of eggs to improve the nutritional status and health outcomes of specified populations.^(3)^ Even though it has been hypothesised that regular provision of eggs to children at risk of undernutrition may prevent stunting, which affects 22% of children under five years old globally and 33% in sub-Saharan Africa,^(4)^ the comparative effectiveness of egg-based interventions against other dietary approaches or no intervention among malnourished children aged 6–59 months in sub-Saharan Africa remains unclear. Moreover, the existing literature presents inconsistent findings. Some studies^(5,6)^ reported an insignificant effect of egg-based intervention on stunting, while others demonstrate notable improvements in child growth indicators following egg-based interventions.^(7–9)^ Furthermore, unlike the previous meta-analysis,^(10)^ which evaluated the impact of egg supplementation on child growth using absolute changes in height/length and weight, our analysis evaluated undernutrition using standardised anthropometric indices, including weight-for-age *z*-score (WAZ), weight-for-height *z*-score (WHZ), and height-for-age *z*-score (HAZ).

Therefore, this meta-analysis is timely for a couple of reasons. First, it provides a comprehensive assessment of the current state of evidence regarding egg-based interventions for malnourished children. Second, the findings inform policymakers, programme planners, and healthcare professionals about the role of egg-based interventions on children’s nutritional status. Third, the review identifies gaps in the existing literature, potentially guiding future studies to deal with child nutrition, health and overall development. Thus, this meta-analysis is conducted to systhmatically synthesize impact of egg-based interventions on child nutritional status as determined by WHZ, WAZ, and HAZ.

## Materials and Methods

This meta-analysis is conducted and reported in accordance with the reporting guidance provided in the Preferred Reporting Items for Systematic Reviews and Meta-Analyses Protocols (PRISMA-P).^(11)^ The study protocol was prospectively registered at PROSPERO (CRD42024537866).

### Eligibility criteria

The population, intervention, comparison, and outcome (PICO) framework guided the study selection process. We included peer-reviewed studies that reported interventions involving egg consumption or egg-based supplementary foods for children, as well as studies assessing compliance with egg consumption interventions in the context of malnutrition.

**Population**: The study population was all children aged 6–59 months old. Studies on children with complicated SAM and any other primary condition affecting child development other than malnutrition, that is, neurological or musculoskeletal disorders, neurodevelopmental disorders, autism spectrum disorders, or cardiovascular (e.g. congenital heart failure), were excluded.

**Intervention**: The study compared the egg/egg-based intervention to any type of dietary intervention or no intervention. Eligible interventions included eggs provided in any form (e.g. boiled, scrambled) as part of the child’s diet. Studies were excluded if they evaluated non-nutritional interventions (e.g. pharmacologic or medical treatments), chicken supplementation rather than egg consumption, acute medical conditions, enteral or parenteral feeding, or therapeutic feeding products used for the treatment of severe acute malnutrition (SAM).

**Comparison**: Studies that compare the egg-based interventions with other dietary interventions (e.g. fortified foods, supplements), behavioural change education, or no intervention at all.

**Outcome**: Studies that report on outcomes related to nutritional status, such as weight gain, height, mid-upper arm circumference (MUAC), height-for-age, weight-for-age, and weight-for-height/length.

**Design and language:** All articles of randomised controlled trials published in English were included. Articles and documents conducted in any other design were excluded, such as observational studies, editorials, conference proceedings, abstracts only, case reports, case series, qualitative formative assessments, discussion papers, gray reports, and unpublished papers.

### Data sources and search strategy

A comprehensive systematic search was conducted across multiple electronic databases, including PubMed/MEDLINE, Web of Science, CINAHL, Embase, Google Scholar, Science Direct, and African Index Medicus (AIM), to identify relevant studies to estimate the egg or egg-based intervention and its effect on malnourished children under five. In addition, reference lists and free web-based searches were conducted to retrieve other relevant materials. The search was performed independently by two authors using the following keywords. The key term used in the database search was ‘egg’ OR ‘egg incorporated’ OR ‘egg enriched’ OR ‘egg-based’ OR ‘egg blended’ OR ‘egg mixed’ OR ‘egg mashed’ AND ‘undernutrition’ OR ‘malnutrition’ OR ‘nutritional status’ OR ‘malnourished’ OR ‘stunting’ OR ‘underweight’ OR ‘wasting’ OR ‘acute malnutrition’ OR ‘HAZ/LAZ’ OR ‘WAZ’ OR ‘WHZ’ OR ‘growth faltering’ AND ‘intervention’ OR ‘treatment’ OR ‘supplement’ OR ‘management’ OR ‘complement’ OR ‘intake’ OR ‘consumption’ AND ‘under five children’ OR ‘infant’ OR ‘young child*’ OR ‘child*’ AND ‘Sub-Saharan Africa’ OR ‘developing country*’ OR ‘low-income country’. Also, a search strategy was developed by combining both MeSH terms and free-text words related to children under five and egg or egg-based food intervention. The search was conducted from April to October 2024 for research articles published between 2013 up to 2023.

### Screening and study selection

All records retrieved from the search were imported into EndNote for deduplication and management. The screening process was conducted in two stages. Two reviewers independently screened all titles and abstracts against the predefined inclusion and exclusion criteria guided by the PICO framework. In the second stage, the same two reviewers independently assessed the full texts of potentially eligible studies. At both stages duplicate (double) screening was performed, and any disagreements were resolved through discussion to reach consensus; a third reviewer was consulted if necessary. Only studies for which both reviewers agreed on inclusion progressed to the next stage. Reasons for exclusion were documented at the full-text review stage. Studies were excluded if the full text was inaccessible after attempts to contact the corresponding author, if the article was not in English, or if key outcome data relevant to the review were not reported. EndNote was used throughout the screening process to organise records, track reviewer decisions, and ensure a systematic and reproducible workflow.

### Data extraction

A data extraction format was developed in a Microsoft Excel spreadsheet. Two authors independently extracted data from the included studies. Disagreements were resolved through discussion. Data on the following interests were extracted from each included research article: first author name, publication year, country where the study was conducted, age of target population, follow-up duration, sample size, study arm (intervention vs control), and mean and standard deviation of both interventional and control groups for baseline and end-line outcomes.

### Outcome variable and measure of effect

The impact of egg-based dietary interventions on children aged 6–59 months was evaluated using three primary outcomes: WHZ, WAZ, and HAZ. According to WHO,^(12)^ these indices are measures of children’s nutritional status. The effect measures used in this review were the mean difference (MD), which is computed by comparing post-intervention measurements (mean and SD). Even though reporting changes from baseline and post-intervention values is recommended, studies rarely report the standard deviation of the change, which makes taking the estimate more complex. According to the Cochrane review guideline,^(13)^ when insufficient information is available in a study to calculate the standard deviation (SD) of change scores, the SDs can be imputed using change-from-baseline SDs for the same outcome measure reported in other studies included in the review. However, the appropriateness of using an SD from another study depends on whether the studies used the same measurement scale, had a similar degree of measurement error, applied the same time interval between baseline and post-intervention assessments, and involved comparable populations. These conditions were not met in our case. To minimise bias associated with imputing SDs from external studies, we applied an alternative approach recommended by Cochrane: comparing post-intervention measurements. In a randomised trial, baseline characteristics are expected to be comparable across groups due to randomisation; therefore, we chose to report the post-intervention means and SDs.

### Risk of bias and quality assessment

Cochrane Risk of Bias tool for randomised trials (RoB 2.0)^(14)^ was used for bias assessment. RoB 2.0 addresses five specific domains: (1) bias arising from the randomisation process; (2) bias due to deviations from intended interventions; (3) bias due to missing outcome data; (4) bias in measurement of the outcome; and (5) bias in selection of the reported result. Two review authors independently applied the tool to each included study and recorded supporting information and justifications for judgements of risk of bias for each domain (low, high, some concerns). Any discrepancies in judgements of risk of bias or justifications for judgements were resolved by discussion to reach consensus between the two review authors, with a third review author acting as an arbiter when necessary.

### Statistical analysis

A narrative synthesis summarised the study characteristics, populations, interventions, outcome measures, and key findings. A random effect Meta-analysis was conducted using Stata software version 17 to estimate the pooled effect of egg-based intervention on the nutritional status of children. Forest plots were used to display the pooled estimates. Heterogeneity was assessed using Cochran’s *Q* statistic, *I* ^2^ statistic, and chi-square test (*p* < 0.05); and the level of heterogeneity was classified as low (*I* ^2^: 0–25%), moderate (*I* ^2^: 25–50%), or high (*I* ^2^: ≥50%).^(15,16)^ Subgroup analyses and meta-regression explored potential sources of heterogeneity, considering study country, duration of the intervention, publication year, and sample size. Publication bias was evaluated using Egger’s test, which quantifies funnel plot asymmetry.^(17,18)^ Sensitivity analysis was also done using the leave-one-out method to evaluate the effect of removing a single study from the analysis.

## Result

### Article screening

**Figure 1.**
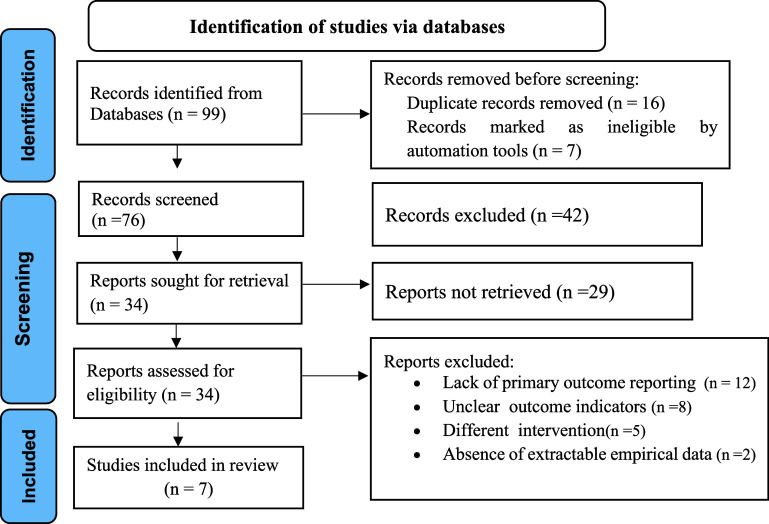
PRISMA flow diagram showing article screening.

A comprehensive search across multiple databases yielded a total of 99 relevant research articles, with 59 from PubMed, 28 from Google Scholar, and 12 from other sources that are listed in the method section. PRISMA flow diagram guided a two-stage article screening work.^(11)^ At the first stage, of the 99 articles retrieved, after reviewing the titles and removing duplicates, 23 articles were excluded. Further scrutiny of the remaining 76 abstracts led to the removal of 42 articles due to abstracts that did not meet the inclusion criteria. At the second stage, the authors then conducted a full-text assessment of the remaining 34 articles; 27 articles were excluded as they did not report their primary outcome (*n* = 12), for being an uncreatable outcome indicator (*n* = 8), a different intervention (*n* = 5), and absence of extractable empirical data (*n* = 2). Finally, 7 articles were included in this SRMA as they met the eligibility criteria (Figure 1).

### Characteristics of the studies

This meta-analysis included seven studies derived from five unique randomised controlled trials conducted in sub-Saharan Africa between 2013 and 2023. Across these trials, a total of 3673 participants were enrolled at baseline, with 1801 assigned to the intervention groups and 1872 to the control groups. All studies employed a randomised controlled trial. The effect of the egg-based intervention on HAZ was examined in 7 studies, on WAZ in 6, and on WHZ in 6. Two studies were included from Malawi,^(5,8)^ one from Kenya,^(9)^ three studies from Ethiopia^(7,19,20)^ and one from South Africa.^(21)^ Three trials^(7,8,19)^ assessed the effect of one egg per day supplementation versus control, and three trials^(5–7)^ assessed the effect of one egg per day supplementation versus behavioural change intervention. Moreover, one trial evaluated the effect of one egg per day supplementation and behaviour-change interventions versus behaviour-change interventions (Table 1).^(9)^

**Table 1.** Characteristics of the studies included in the review

Author	Child age at enrolment (months)	Country	Duration of intervention (months)	Sample size intervention	Sample size control	Intervention	Quality of the evidence
Stewart et al., 2019^(8)^	6–9	Malawi	6	290	305	1 egg/d vs no intervention	Medium
Wegmüller et al., 2022^(9)^	0–35	Kenya	24	375	361	1 egg vs Behavioural education	High
Omer et al., 2022^(7)^	6–18	Ethiopia	6	122	121	1 egg/day vs behavioural education	Medium
Passarelli et al., 2020^(19)^	0–35	Ethiopia	18	242	307	1 egg vs no intervention	High
Ricci et al., 2023^(21)^	6–9	South Africa	6	250	250	1 egg/day vs behavioural education	Medium
Anteneh Omer et al., 2019^(20)^	6–12	Ethiopia	6	122	128	1 egg and 1 eggshell powder/day vs no intervention	Medium
Bragg et al., 2023^(5)^	6–9	Malawi	6	400	400	1 egg/day vs behavioural education	High

### Risk of bias assessment

We used the RoB 2.0 tool to assess the risk of bias for each of the included studies. All the studies included in the review had a low risk of bias arising from the randomisation process. In one study,^(7)^ the participants were not blinded to the intervention, and two of the studies did not report whether participants and/or personnel were blinded, resulting in an unclear risk of bias.^(19, 21)^ And only the assessor was blinded,^(8)^ and there is no information on blinding.^(20)^ Regarding bias due to missing outcome data, a 10% loss to follow-up was reported from one study,^(8)^ and even though the final sample had 80% power, a 25% lost from the study^(5)^ had a high risk of bias. Almost all studies had a low risk of bias in the measurement of the outcome, in which they used appropriate outcome measurement. However, one study^(21)^ didn’t report the outcome to measure weight for age and weight for height. Overall, one study was identified as high risk due to a lack of information on allocation sequence, either adequately concealed or not, lack of blinding of both participants, assessor, and a higher loss to follow-up rate.^(5)^ Studies such as^(7,19,21)^ had some concerns about risk, and studies^(8,9,20)^ had a low risk of bias.

### The impact of egg-based interventions on weight-for-age

**Figure 2.**
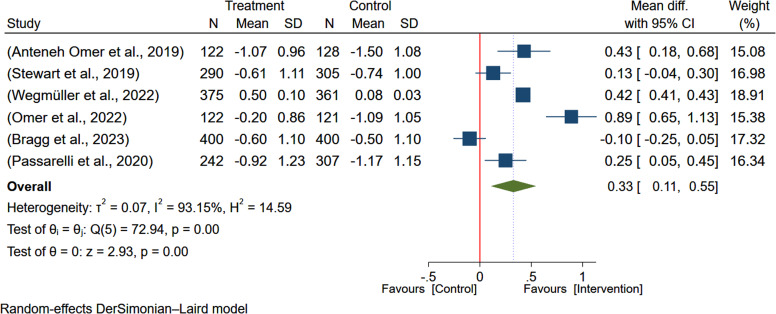
Pooled effect of egg-based interventions on weight-for-age *z*-scores (WAZ) among children aged 6–59 months.

Six studies^(5,7-9,19,20)^ encompassing 3173 participants were analysed to evaluate the effect of egg-based interventions on the weight-for-age of the children. Due to heterogeneity between studies (*I* ^2^ = 93.13%), a random-effects meta-analysis was employed to pool the effect sizes. The results indicated that egg-based intervention significantly improved the weight-for-age of the children (MD: 0.33; 95% CI: 0.11–0.55) (Figure 2).

#### Investigating heterogeneity

**Figure 3.**
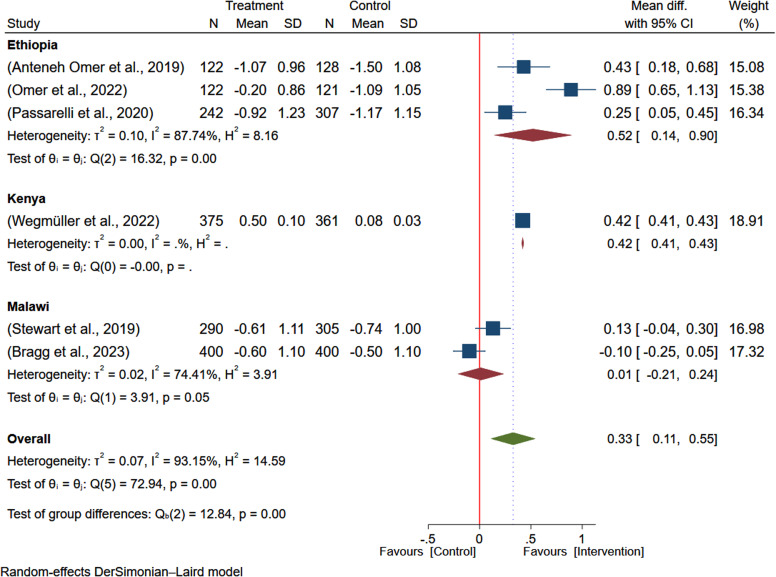
Subgroup analysis of the pooled effect of egg-based interventions on weight-for-age *z*-scores (WAZ) among children aged 6–59 months, stratified by country.

**Figure 4.**
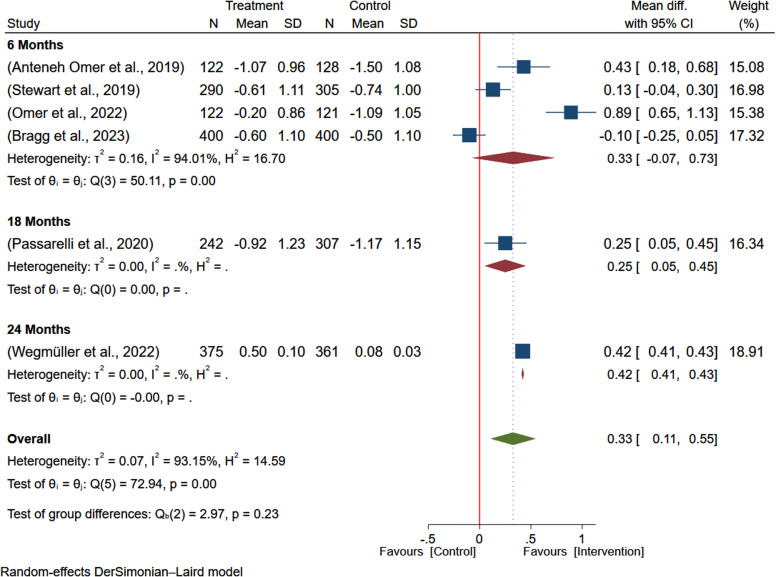
Subgroup analysis of the pooled effect of egg-based interventions on weight-for-age *z*-scores (WAZ) among children aged 6–59 months, stratified by duration of intervention.

To investigate sources of heterogeneity, subgroup analysis by country of the study and duration of intervention was conducted. Based on the subgroup analysis, the effect size in Ethiopia (MD: 0.52; 95% CI: 0.14–0.90) and Kenya (MD: 0.42; 95% CI: 0.41–0.43) indicates the study country may significantly contribute to the statistical heterogeneity. However, the effect size from studies from Malawi (MD: 0.13; 95% CI: –0.04–0.30) was not significant (Figure 3). The subgroup analysis based on the duration of intervention, the overall estimate was higher among studies with longer duration of intervention (24 months) reporting larger effect of intervention on the WAZ (MD: 0.42; 95% CI: 0.41–0.43) and, studies with intervention duration of 6 months reported non-significant effect size (MD: 0.33; 95% CI: –0.07-0.73) (Figure 4).

The meta-regression analysis on the effect of egg-based interventions on children’s WAZ yielded inconclusive results. None of the moderators (intervention group size, control group size, and publication year) showed statistically significant effects (all *p*-values > 0.05). These findings suggest that other factors, not included in this analysis, may be more important in explaining the variability in the effects of egg-based interventions on children’s WAZ scores. The statistical heterogeneity may be due to unmeasured factors and factors analysed in subgroup analysis, such as countries and intervention duration.

#### Assessment of publication bias

**Figure 5.**
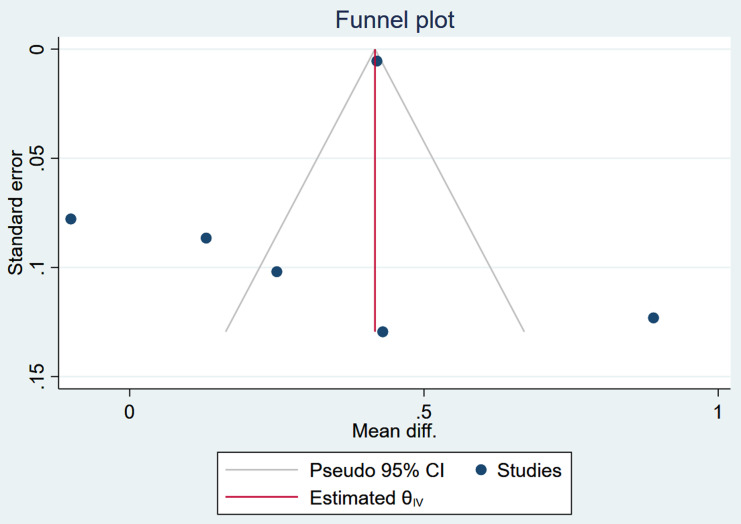
Result on publication bias assessment of studies included in the meta-analysis of egg-based interventions on weight-for-age *z*-scores (WAZ).

Publication bias was assessed using the funnel plot and Egger’s test (*β* =1.7, *p* = 0.6298), which provided a non-significant regression intercept, indicating no evidence of the small-study effects and evidence of publication bias. The funnel plot indicates a slight deviation of the plot to the left and a suggestion of publication bias (Figure 5). However, trim and fill analysis indicated that additional studies are needed. This indicates the publication bias is not a major issue in the analysis.

#### Sensitivity analysis

**Figure 6.**
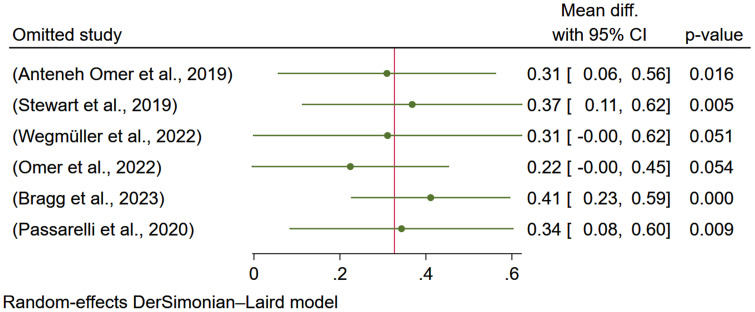
Sensitivity analysis (leave-one-out) showing the effect of egg-based interventions on weight-for-age *z*-score (WAZ) with 95% CIs.

The sensitivity analysis indicated that removing two studies^(7, 9)^ affects the overall estimate of the effect of egg-based interventions on weight-for-age. Removing the study done by Omer et al.^(7)^ reduces the estimate to 0.22 and makes it negligible. While removing the study done by Wegmüller et al.^(9)^ reduces the effect size to 0.31 and is insignificant (Figure 6).

### The impact of egg-based interventions on weight-for-height

**Figure 7.**
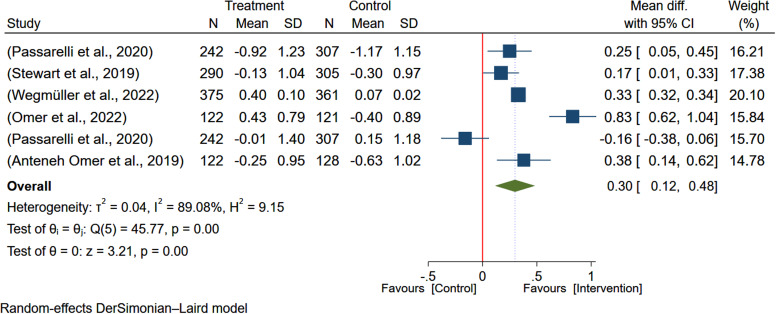
Pooled effect of egg-based interventions on weight-for-height *Z*-scores (WHZ) among children aged 6–59 months.

Six studies^(5,7–9,19,20)^ with a total of 3173 participants were analysed to evaluate the effect of egg-based interventions on the weight-for-height. Due to heterogeneity between studies (*I* ^2^ = 89.39%), a random-effects meta-analysis was employed to pool the effect sizes. The results indicated that egg-based intervention significantly improved weight-for-height (MD: 0.30; 95% CI: 0.12–0.48) (Figure 7).

#### Investigating heterogeneity

**Figure 8.**
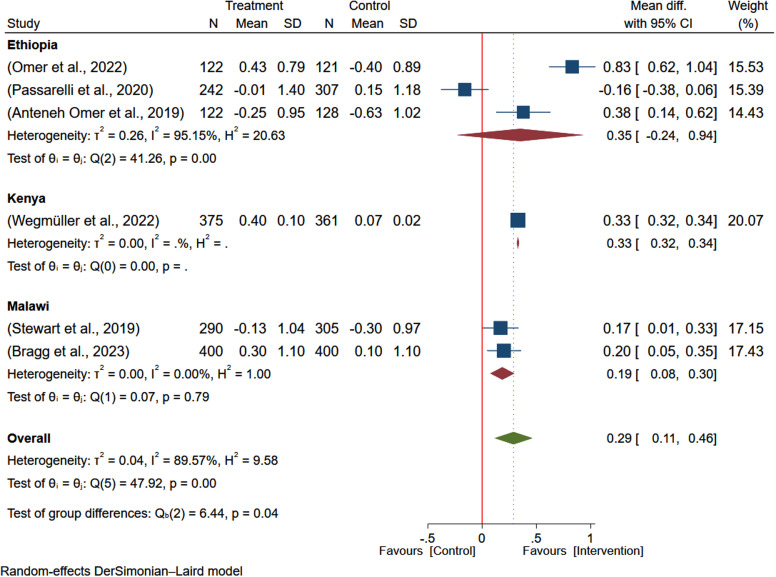
Subgroup analysis of the pooled effect of egg-based interventions on weight-for-height *Z*-scores (WHZ), among children aged 6–59 months. stratified by study country.

**Figure 9.**
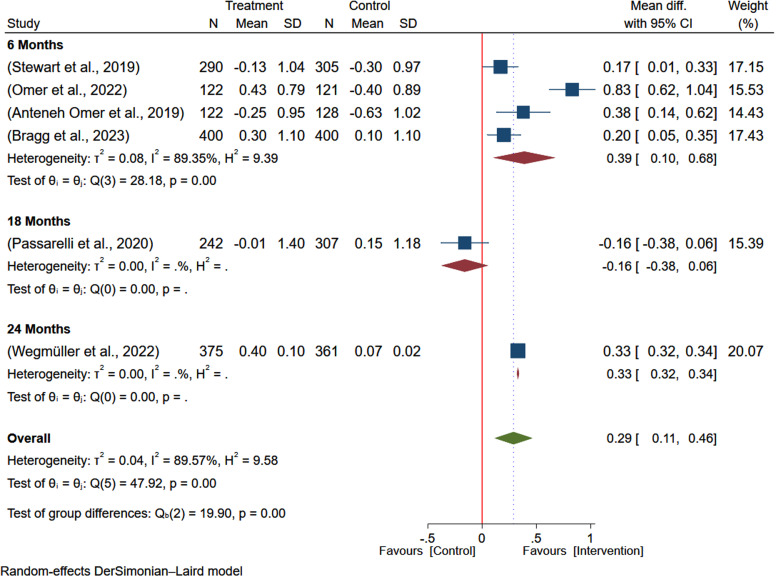
Subgroup analysis of the pooled effect of egg-based interventions on weight-for-height *Z*-scores (WHZ) among children aged 6–59 months, stratified by intervention duration.

Based on the subgroup analysis by country, the effect of egg-based interventions on weight-for-height (WHZ) showed some variation. Studies in Ethiopia reported a non-significant positive effect (MD: 0.35; 95% CI: –0.23–0.93), whereas the findings from Kenya (MD: 0.33; 95% CI: 0.32–0.34) and Malawi (MD: 0.19; 95% CI: 0.07–0.30) were significant (Figure 8). The estimate was higher among studies with longer duration of intervention (24 months) reporting a larger effect of intervention on the WHZ (MD: 0.33; 95% CI: 0.32–0.34) (Figure 9).

Moreover, a meta-regression analysis examining the effect of egg-based interventions on children’s weight-for-height revealed significant moderating effects of sample sizes and a potential influence of publication year. The analysis showed that larger intervention group sample sizes were associated with greater positive effects (coefficient = 0.0059602, *p* = 0.002), while larger control group sizes were linked to smaller effects (coefficient = -0.0080304, *p* = 0.000). However, publication year was a non-significant trend with no effect on the pooled estimate of the egg-based intervention on WHZ (coefficient = 0.0575818, *P* = 0.084). Thus, the model explained 95.82% of the variability in effect sizes, with low to moderate heterogeneity (*I* ^2^ = 24.22%). The analysis was limited by the small number of studies and the potential influence of unmeasured moderators, suggesting avenues for future research to enhance our understanding of factors affecting the efficacy of egg-based interventions in improving children’s nutritional status.

#### Assessment of publication bias

**Figure 10.**
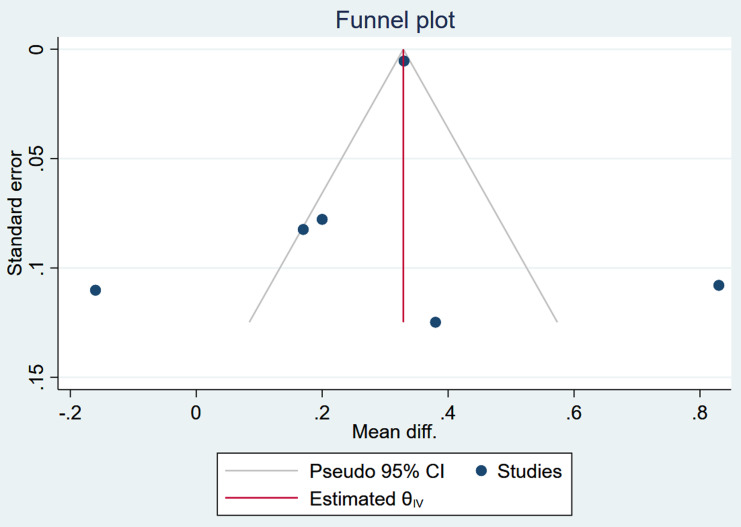
Result on publication bias assessment of studies included in the meta-analysis of egg-based interventions on Weight-for-height *Z*-scores (WHZ).

Publication bias was assessed using the funnel plot and Egger’s test. Therefore, Egger’s test (*B* = 0.1, *P* = 0.9741) provided a non-significant regression intercept, indicating no evidence of the small-study effects and evidence of publication bias. The funnel plot indicates a slight deviation of the plot to the left and a suggestion of publication bias (Figure 10). However, the trim and fill analysis suggests the presence of publication bias in the meta-analysis, with two potentially missing studies imputed. After adjustment, the estimated MD increased from 0.290 (95% CI: 0.116–0.464) to 0.423 (95% CI: 0.240–0.606), indicating that the original analysis may have underestimated the intervention’s effect. While this suggests a potentially stronger intervention effect, the results should be interpreted cautiously due to the limitations of the trim and fill method and the small number of observed studies. Egger’s tests indicate no publication bias, while the trim-and-fill method suggests its presence. This could be because Egger’s tests often have low statistical power, especially with a small number of studies.

#### Sensitivity analysis

**Figure 11.**
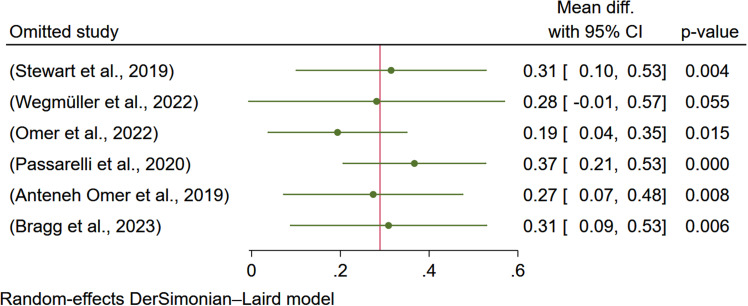
Sensitivity analysis (leave-one-out) showing the effect of egg-based interventions on Weight-for-height *Z*-score (WHZ) with 95% CIs.

The sensitivity analysis indicated that the study done by Wegmüller et al.^(9)^ affects the pooled estimate of the effect of egg-based interventions on weight-for-height and makes it negligible (Figure 11).

### The impact of egg-based interventions on height-for-age

**Figure 12.**
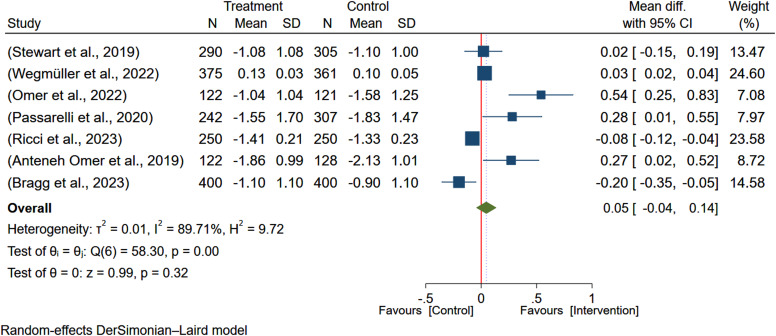
Pooled effect of egg-based interventions on height-for-age *z*-scores (HAZ) among children aged 6–59 months.

Seven studies^(5, 7–9, 19–21)^ encompassing 3673 participants were analysed to evaluate the effect of egg-based interventions on the height-for-age of the children. Due to heterogeneity between studies (*I* ^2^ = 89.68%), a random-effects meta-analysis was employed to pool the effect sizes. The results indicated that egg-based intervention did not significantly improve the height-for-age of the children (MD: 0.05; 95% CI: –0.05–0.14) (Figure 12).

#### Investigating heterogeneity

Based on subgroup analysis, while studies in Ethiopia (MD: 0.35; 95% CI: 0.19–0.52) and Kenya (MD: 0.03; 95% CI: 0.02–0.03) showed promise, others in those countries and studies from Malawi and South Africa produced mixed results. High heterogeneity across studies (except in Ethiopia) suggests these findings may not be generalisable. The subgroup analysis done using the duration of intervention shows that the longer the duration of intervention, the higher the effect of intervention on height-for-age. Thus, the intervention duration of 24 months had a significantly higher effect of egg-based intervention (MD: 0.03; 95% CI: 0.02–0.04).

The meta-regression revealed that none of the moderators (intervention group size, control group size, and publication year) showed statistically significant effects (all *p*-values > 0.05). These findings suggest that other factors, not included in this analysis, may be more important in explaining the variability in the effects of egg-based interventions on children’s HAZ scores. The statistical heterogeneity may be due to unmeasured factors and factors analysed in subgroup analysis, such as countries and intervention duration.

Publication bias was assessed using a regression-based Egger test under a random-effects model. The test indicated significant small-study effects (*β*_1_ = 2.57, *p* = 0.011), suggesting potential publication bias. However, the trim-and-fill analysis did not impute any missing studies, suggesting that the observed asymmetry may not materially affect the pooled estimate.

Sensitivity analysis done using the leave-one-out method and removing single study turn by turn didn’t affect the pooled estimate of the effect of egg-based intervention on the height-for-age of the children.

## Discussion

This meta-analysis pooled the collective effect of egg-based interventions compared to other alternative interventions or no intervention among children aged 6–59 months in SSA. It figuredout that egg-based interventions can help enhance weight-related nutritional outcomes (WAZ and WHZ) of children aged 6–59 months.

The meta-analysis revealed a noteworthy beneficial impact of egg-based interventions on Weight-for-Age (WAZ) scores (MD: 0.33) and Weight-for-Height (WHZ) (MD: 0.29), indicating that adding eggs to the diet could contribute to child weight increment. This finding is consistent with other previous reports done on the effect of egg on the weight of children,^(10)^ which reported that egg included in the diet had a positive impact on weight. Similarly, a report from China^(22)^ and low-income countries likely to be resource limited^(23,24)^ indicated that the egg intervention can improve child development. This could be for that egg is rich in complete protein and other important micronutrients. An average egg contains approximately 75 calories, 7 g protein, 5 g fat, 1.6 g saturated fat, vitamins, and carotenoids^(25)^; and egg intervention increased the quantity and quality of protein intakes among young children.^(26)^ This review indicated that studies conducted in Ethiopia (MD 0.52) reported the highest MD, indicating the effect of egg-based intervention was most important. The length of the intervention seemed to impact WAZ and WHZ outcomes, as longer interventions (24 months) had greater effects on WAZ (MD: 0.42) and WHZ (MD: 0.33), compared to shorter interventions (6 months) with insignificant effects. Also, a similar positive result was reported by a research from Bangladish^(27)^ in which daily intake of eggs for a longer time can have a significant impact on the linear growth of the children. Eggs have been suggested as a potentially efficacious dietary intervention because of their nutrient density, as they contain all the essential aminoacids forming complete protein, vitamins, and minerals that are significant to child growth and development.^(13,25)^ Eggs are a rich source of multiple nutrients that support growth and development among young children, including protein with an optimal balance of amino acids to support requirements, and vitamins and minerals such as vitamin A, vitamin B12, choline, and selenium.^(13,28)^ Eggs are recognised for their high nutrient density, which supports growth and development, particularly in resource-limited settings.^(6)^ The continual discovery that extending intervention lengths resulted in greater impacts highlights the necessity for ongoing, extended nutrition initiatives. However, while the egg remains a nutritional powerhouse, its effectiveness is mediated by the complex realities of biology, environment, and existing diet.^(29)^ When developing and carrying out interventions centred on eggs, it is important to take into account factors like the cultural acceptance of eggs, local food systems, and the existing nutritional status of the population.^(30)^ Although subgroup analysis found a non-significant intervention effect on WHZ in Ethiopia, this aligns with prior evidence: a recent systematic review of child nutrition programmes in Ethiopia reported that nearly two-thirds of interventions had no impact on stunting or wasting, and more than half failed to improve underweight.^(31)^ A more recent review of food and nutrition programmes (2015–2025) also observed that single-component interventions often produced limited or inconsistent improvements in child anthropometry, while multicomponent programmes (e.g. combining supplementation, food support, maternal education, WASH, and social protection) were more likely to yield positive results.^(32)^ These findings together suggest that the lack of statistically significant effect in our Ethiopian sample may reflect structural and contextual constraints such as inadequate dietary diversity, insufficient programme intensity or duration, weak integration of complementary interventions, or high baseline burden of malnutrition rather than a failure of the overall concept of nutritional intervention. It highlights the need for future interventions in Ethiopia to adopt a comprehensive, integrated approach (combining nutrition, WASH, food security, maternal education, and social protection) rather than relying on a single component to effectively reduce undernutrition.

However, egg-based interventions did not have a significant impact on height-for-age (HAZ) scores, with an MD of 0.05. This indicates that egg-focused strategies may not significantly affect height development in the near to mid-term. The finding is consistent with the report from Bangladesh.^(24)^ The intervention reported did not have a statistically significant effect on mean LAZ, but it did have a significant impact on the WHZ. This could be the fact that chronic undernutrition and stunting are complex issues influenced by multiple factors, including overall dietary diversity, health status, and environmental conditions.^(10)^ Furthermore, the substantial diversity among studies (*I* ^2^ = 89.68%) shows significant variation in results, which could be due to disparities in study countries or implementation strategies. When subgroup analysis was conducted by country, Ethiopia (MD: 0.35) showed positive outcomes. Moreover, longer intervention length was identified as a possibly crucial element, as extended interventions (24 months) had a notable beneficial impact on HAZ. The variation in geography on the effect of egg-based intervention on stunting indicates that the success of egg-related interventions could depend on the specific situation, potentially affected by factors like eating habits, cultural acceptance of eggs, local food systems, and the existing nutritional status of the population or surroundings.^(10)^ Moreover, continued nutritional interventions may be needed to make significant enhancements in height gain.^(33)^

### Strength

The primary strength of this study lies in its power to provide a definitive, evidence-based answer to a critical public health question using the most rigorous methodological approach available. It moves beyond anecdote or findings from a single location to offer a consolidated, quantitative verdict on the role of eggs in combating child malnutrition in a high-need region. This makes it an exceptionally valuable tool for researchers, policymakers, and practitioners aiming to implement effective, scalable nutrition interventions.

### Limitations and future research directions

The high heterogeneity observed across studies for all outcomes suggests that unmeasured factors may influence the effectiveness of egg-based interventions. Future research should aim to identify and quantify these factors. Future research should investigate the long-term impacts of egg-based interventions on child growth, development, and health outcomes. The analysis did not consider the cost-effectiveness of egg-based interventions compared to other nutrition strategies. Future research should incorporate economic evaluations to inform policy decisions. Furthermore, the subgroup analysis included a limited number of studies, which may not reliably reflect true differences in effect sizes across countries or intervention durations. To address the limitations of current interventional researches, future studies should evaluate the efficacy of egg-based interventions and the specific timeframe required to achieve measurable outcomes in malnourished children.

## Conclusion

This meta-analysis demonstrates that egg-based interventions can improve weight-related nutritional outcomes (WAZ and WHZ) in children aged 6–59 months across sub-Saharan Africa, particularly weight-for-age WAZ) and weight-for-height (WHZ) Z-scores. These findings suggest that incorporating eggs into children’s diets can contribute to their physical growth. The strongest positive effect of egg-intervention on child physical growth was observed in Ethiopia. Moreover, impact of egg-based intervention on child growth depends length of the intervention, longer intervention duration resulted with agreator positive impact. Nevertheless, the absence of notable impacts on linear growth/height (HAZ) indicates that adding eggs by themselves may not be enough to tackle chronic undernutrition. The impact of these measures seems to be affected by elements like location, length of intervention, and scope of implementation. Further, future studies should concentrate on enhancing intervention strategies focusing on evaluating the effect of egg-based interventions among children with malnutrition, examining the length of intervention on the children’s anthropometric outcomes, and delving into cost efficiency and durability evaluations. Policymakers and programme implementers should incorporate egg-based nutrition interventions in nutrition programs to support children in resource limited settings grow and develop to their full potential growth falterings.

## References

[1] WHO. Levels and trends in child malnutrition: UNICEF/WHO/The World Bank Group joint child malnutrition estimates: key findings of the 2021 edition. World Health Organization. 2021.

[2] UNICEF, WHO, World Bank. The UNICEF, WHO and the World Bank inter-agency team update the joint global and regional estimates of malnutrition among children under 5 years every other year. Online: UNICEF. 2025. https://data.unicef.org/resources/jme/.

[3] Van den Heuvel E , Murphy JL , Appleton KM. Towards a food-based intervention to increase protein intakes in older adults: challenges to and facilitators of egg consumption. Nutrients. 2018;10(10):1409. 30279360 10.3390/nu10101409 PMC6213861

[4] UNICEF. The State of the World’s children 2019. Children, food and nutrition: growing well in a changing world. New York: UNICEF. 2019.

[5] Bragg MG , Prado EL , Caswell BL , et al. The association between plasma choline, growth and neurodevelopment among Malawian children aged 6-15 months enroled in an egg intervention trial. Matern Child Nutr. 2023;19(2):e13471. 36567549 10.1111/mcn.13471 PMC10019050

[6] Ricci H , Faber M , Ricci C , et al. Effects of egg as an early complementary food on growth of 6- to 9-month-old infants: a randomised controlled trial. Public Health Nutr. 2024;27(1):e1. 10.1017/S1368980023002604 PMC10830362 38018158

[7] Omer A , Hailu D , Whiting SJ. Effect of a child-owned poultry intervention providing eggs on nutrition status and motor skills of young children in southern Ethiopia: a cluster randomized and controlled community trial. Int J Env Res Pub He. 2022;19(22):15305. 10.3390/ijerph192215305 PMC9690635 36430025

[8] Stewart CP , Caswell B , Iannotti L , et al. The effect of eggs on early child growth in rural Malawi: the Mazira Project randomized controlled trial. Am J Clin Nutr. 2019;110(4):1026–1033. 31386106 10.1093/ajcn/nqz163 PMC6766435

[9] Wegmüller R , Musau K , Vergari L , et al. Effectiveness of an integrated agriculture, nutrition-specific, and nutrition-sensitive program on child growth in Western Kenya: a cluster-randomized controlled trial. Am J Clin Nutr. 2022;116(2):446–459. 35421217 10.1093/ajcn/nqac098 PMC9348977

[10] Larson EA , Zhao Z , Bader-Larsen KS , Magkos F. Egg consumption and growth in children: a meta-analysis of interventional trials. Front Nutr. 2023;10:1278753. 38249601 10.3389/fnut.2023.1278753 PMC10796599

[11] Page MJ , McKenzie JE , Bossuyt PM , et al. The PRISMA. 2020 statement: an updated guideline for reporting systematic reviews. Int J Surg. 2021;88:105906. 33789826 10.1016/j.ijsu.2021.105906

[12] WHO. Assessing and managing children at primary health-care facilities to prevent overweight and obesity in the context of the double burden of malnutrition: updates for the integrated management of childhood illness (IMCI). Table 1, World Health Organization (WHO) classification of nutritional status of infants and children Geneva: World Health Organization. 2017. 29578661

[13] Larson EA , Zhao Z , Bader-Larsen KS , Magkos F. Egg consumption and growth in children: a meta-analysis of interventional trials. Front Nutr. 2024;10:1278753. 38249601 10.3389/fnut.2023.1278753 PMC10796599

[14] Higgins JP , Altman DG , Gøtzsche PC , et al. The Cochrane Collaborations tool for assessing risk of bias in randomised trials. BMJ. 2011;343:d5928. 22008217 10.1136/bmj.d5928 PMC3196245

[15] Higgins JP , Thompson SG. Quantifying heterogeneity in a meta-analysis. Stat Med. 2002;21(11):1539–1558. 12111919 10.1002/sim.1186

[16] Cochran WG. The combination of estimates from different experiments. Biometrics. 1954;10(1):101–129.

[17] Egger M , Smith GD , Schneider M , Minder C. Bias in meta-analysis detected by a simple, graphical test. BMJ. 1997;315(7109):629–634. 9310563 10.1136/bmj.315.7109.629 PMC2127453

[18] Begg CB , Mazumdar M. Operating characteristics of a rank correlation test for publication bias. Biometrics. 1994;50:1088–1101. 7786990

[19] Passarelli S , Ambikapathi R , Gunaratna NS , et al. A chicken production intervention and additional nutrition behavior change component increased child growth in Ethiopia: a cluster-randomized trial. J Nutr. 2020;150(10):2806–2817. 32652012 10.1093/jn/nxaa181 PMC7549301

[20] Omer A , Mulualem D , Classen H , Vatanparast H , Whiting SJ. Promotion of egg and eggshell powder consumption on the nutritional status of young children in Ethiopia. Int J Food Sci Nutr Res. 2019;1:1004.

[21] Ricci H , Faber M , Ricci C , et al. Effects of egg as an early complementary food on growth of 6- to 9-month-old infants: a randomised controlled trial. Public Health Nutr. 2023;27(1):e1. 38018158 10.1017/S1368980023002604 PMC10830362

[22] Chen J-H , Jin M. The effectiveness of an egg-based intervention on improving the nutrition of poor school-age children in China: a quasi-experimental assessment. Nutrition. 2023;109:111994. 36905839 10.1016/j.nut.2023.111994

[23] Iannotti LL , Lutter CK , Bunn DA , Stewart CP. Eggs: the uncracked potential for improving maternal and young child nutrition among the world’s poor. Nutr Rev. 2014;72(6):355–368. 24807641 10.1111/nure.12107

[24] Pasqualino MM , Shaikh S , Hossain MI , et al. An egg intervention improves ponderal but not linear growth among infants 6-12 mo of age in Rural Bangladesh. J Nutr. 2024;154(7):2290–2299. 38759886 10.1016/j.tjnut.2024.05.006

[25] Lutter CK , Iannotti LL , Stewart CP. The potential of a simple egg to improve maternal and child nutrition. Matern Child Nutr. 2018;14:e12678. 30332538 10.1111/mcn.12678 PMC6865885

[26] Caswell B , Arnold C , Lutter C , Maleta K , Stewart C. An egg feeding intervention increased protein quantity and quality among young Malawian children. Curr Dev Nutr. 2020; 4:nzaa054_27.

[27] Mahfuz M , Alam MA , Das S , et al. Daily supplementation with egg, cow milk, and multiple micronutrients increases linear growth of young children with short stature. J Nutr. 2020;150(2):394–403. 31665385 10.1093/jn/nxz253

[28] Iannotti LL , Lutter CK , Stewart CP , et al. Eggs in early complementary feeding and child growth: a randomized controlled trial. Pediatrics. 2017;140(1):3–5. 10.1542/peds.2016-3459 28588101

[29] Stewart CP. Has the golden egg lost its luster? Am J Clin Nutr. 2025;122(5):1153–1154. 41093697 10.1016/j.ajcnut.2025.09.021

[30] Blum LS , Swartz H , Olisenekwu G , Erhabor I , Gonzalez W. Social and economic factors influencing intrahousehold food allocation and egg consumption of children in Kaduna State, Nigeria. Matern Child Nutr. 2023;19(1):e13442. 36353982 10.1111/mcn.13442 PMC9749605

[31] Ahmed KY , Ogbo FA , Tegegne TK , Dalton H , Arora A , Ross AG. Interventions to improve the nutritional status of children under 5 years in Ethiopia: a systematic review. Public Health Nutr. 2023;26(12):3147–3161. 37905557 10.1017/S1368980023002410 PMC10755407

[32] Ayele K , Demisew M , Gemede HF. A systematic review of the impact of food and nutrition programs on child nutrition and household food security in Ethiopia. BMC Nutrition. 2025;11(1):214. 41239539 10.1186/s40795-025-01194-z PMC12619373

[33] Siegal K , Wekesa B , Custer E , Gatwaza TH , Uweh J , Niyonshuti M. A good egg: an evaluation of a social and behavior change communication campaign to increase egg consumption among children in Rwanda. Matern Child Nutr. 2024;20(1):e13573. 37830401 10.1111/mcn.13573 PMC10750004

